# Neuromuscular electrical stimulation acutely mobilizes endothelial progenitor cells in critically ill patients with sepsis

**DOI:** 10.1186/s13613-016-0123-y

**Published:** 2016-03-11

**Authors:** Christos Stefanou, Eleftherios Karatzanos, Georgios Mitsiou, Katerina Psarra, Epameinondas Angelopoulos, Stavros Dimopoulos, Vasiliki Gerovasili, Efstathios Boviatsis, Christina Routsi, Serafeim Nanas

**Affiliations:** 1st Critical Care Department, Evangelismos General Hospital, School of Medicine, National and Kapodistrian University of Athens, 45-47 Ypsilantou Str., 106 75 Athens, Greece; Immunology and Histocompatibility Department, Evangelismos General Hospital, 45-47 Ypsilantou Str, 106 75 Athens, Greece; Critical Care Unit, Guys and St Thomas Hospital, Westminster Bridge Road, London, SE1 7EH UK; 2nd Neurosurgical Department, Attiko University General Hospital, School of Medicine, National and Kapodistrian University of Athens, 1 Rimini Str, 124 62 Athens, Greece

**Keywords:** Electrical muscle stimulation, NMES, EPC, Early rehabilitation, Critical illness, Acute effect

## Abstract

**Background:**

Endothelial progenitor cells (EPCs) have been suggested to constitute a restoration index of the disturbed endothelium in ICU patients. Neuromuscular electric stimulation (NMES) is increasingly employed in ICU to prevent comorbidities such as ICU-acquired weakness, which is related to endothelial dysfunction. The role of NMES to mobilize EPCs has not been investigated yet. The purpose of this study was to explore the NMES-induced effects on mobilization of EPCs in septic ICU patients.

**Methods:**

Thirty-two septic mechanically ventilated patients (mean ± SD, age 58 ± 14 years) were randomized to one of the two 30-min NMES protocols of different characteristics: a high-frequency (75 Hz, 6 s on–21 s off) or a medium-frequency (45 Hz, 5 s on–12 s off) protocol both applied at maximally tolerated intensity. Blood was sampled before and immediately after the NMES sessions. Different EPCs subpopulations were quantified by cytometry markers CD34^+^/CD133^+^/CD45^−^, CD34^+^/CD133^+^/CD45^−^/VEGFR_2_^+^ and CD34^+^/CD45^−^/VEGFR_2_^+^.

**Results:**

Overall, CD34^+^/CD133^+^/CD45^−^ EPCs increased from 13.5 ± 10.2 to 20.8 ± 16.9 and CD34^+^/CD133^+^/CD45^−^/VEGFR_2_^+^ EPCs from 3.8 ± 5.2 to 6.4 ± 8.5 cells/10^6^ enucleated cells (mean ± SD, *p* < 0.05). CD34^+^/CD45^−^/VEGFR_2_^+^ EPCs also increased from 16.5 ± 14.5 to 23.8 ± 19.2 cells/10^6^ enucleated cells (mean ± SD, *p* < 0.05). EPCs mobilization was not affected by NMES protocol and sepsis severity (*p* > 0.05), while it was related to corticosteroids administration (*p* < 0.05).

**Conclusions:**

NMES acutely mobilized endothelial progenitor cells, measures of the endothelial restoration potential, in septic ICU patients.

## Background

Endothelium, a key regulator of homeostasis, is disturbed in ICU patients and is associated with multi-organ failure [1]. The relation of endothelial function with endothelial progenitor cells (EPCs) has been shown for different groups, such as healthy populations with or without cardiovascular risk factors [2, 3], patients with coronary artery disease [4] and chronic heart failure (CHF) [5]. Accumulating evidence has suggested multiple roles for EPCs in endothelial physiology, such as neovascularization, endothelial repair and restoration of endothelial function [6]. EPCs are bone marrow-derived precursors of endothelial cells with potential capacity to proliferate, migrate, differentiate or exert paracrine action.

Neuromuscular electrical stimulation (NMES), suggested as an alternative form of exercise in critically ill patients, seems to be potentially beneficial in preserving muscle strength and muscle mass as well as in reducing the duration of weaning and the incidence of ICU-acquired weakness [7–10]. Acutely applied NMES in ICU also induced systemic beneficial effects on the microcirculation, which is closely related to endothelial function [11]. Furthermore, increased EPCs levels have been observed in response to acute bouts of exercise in healthy populations [12–14] and patients with CHF [5]. However, the role of acute NMES in raising EPCs counts in ICU patients has not been investigated yet.

It was hypothesized that an NMES session would increase counts of EPCs in severe ICU patients. The main purpose of this study was to explore the role of acutely applied NMES on mobilizing progenitor endothelial cells in septic critically ill patients.

## Methods

### Patients

Patients admitted to the multi-disciplinary ICUs of Evangelismos Athens General Hospital were considered for inclusion. Inclusion criteria were the presence of sepsis for >72 h and mechanical ventilation. Determination of sepsis was based on criteria, as previously described [15]. Exclusion criteria were age <18 and >80 years, pregnancy, moribund, body mass index (BMI) >35 kg/m^2^, severe edema (deep indentation when pressing finger into skin, requiring >30 s to rebound), ICU-acquired weakness, ICU length of stay ≥20 days prior to study enrollment, history of neuromuscular disease at admission, implanted pacemaker/defibrillator, technical restrictions not allowing NMES application (e.g., bone fractures or skin lesions), thromboembolic disease, continuous hemodialysis, ongoing transfusion, and chemotherapy or other forms of myelotoxicity.

### Study design

This was a prospective randomized study. Eligible patients were randomized to one session of NMES using one of the two protocols with different characteristics. Blood samples were collected before and within 5 min after the session for flow cytometry analyses. Mean arterial blood pressure, heart rate, respiratory rate and ECG were monitored during the session. Lactate and creatine phosphokinase were also recorded before and after the NMES application. Primary end-point was the NMES effect on the number of EPCs. Other end-points were the effects of different NMES protocols, corticosteroids administration and sepsis severity on EPCs number. For the purpose of the latter end-point, patients were divided into two subgroups based on the median SOFA score on the NMES session day (lower SOFA score group ≤6; higher SOFA score group >6).

The study was conducted in accordance with the Declaration of Helsinki and approved by the ethics committee of the hospital. Written informed consent was obtained by the patients or their next of kin.

### Application of neuromuscular electrical stimulation

For NMES application (Rehab 4 Pro, CEFAR Medical AB, Malmö, Sweden), rectangular electrodes (90 × 50 mm) were placed on the motor points of vastus lateralis, vastus medialis and peroneus longus of both lower extremities, after appropriate skin preparation. During the session, the angle of the patients’ knee joint was approximately 40° (0° corresponding to full knee extension). NMES sessions lasted for 40 min including 10 min for warm-up and recovery (10 Hz, 400 μs).

Patients were assigned to a high-frequency (HF) or a medium-frequency protocol (MF), as previously described [16]. In short, both protocols employed symmetric, biphasic, trapezoid pulses, with 400-μs pulse duration and 1.5/0.8-s ramp-up/ramp-down duration, respectively. HF consisted of 75 Hz pulses with 6 s on–21 s off. MF consisted of 45 Hz pulses with 5 s on–12 s off. Current intensity, optimally aiming at full muscle contraction, was continuously increased during the sessions, to prevent fatigue. In sedated patients, starting intensity was set at 60–80 % of that resulting in maximal response and increased by 10 % every 3 min up to 100 %. In non-sedated patients, intensity was initially set to the maximum tolerated level and was increased by 10 % (or less in case of discomfort) every 3 min throughout the session.

### Blood sampling and flow cytometry analyses

**Fig. 1:**
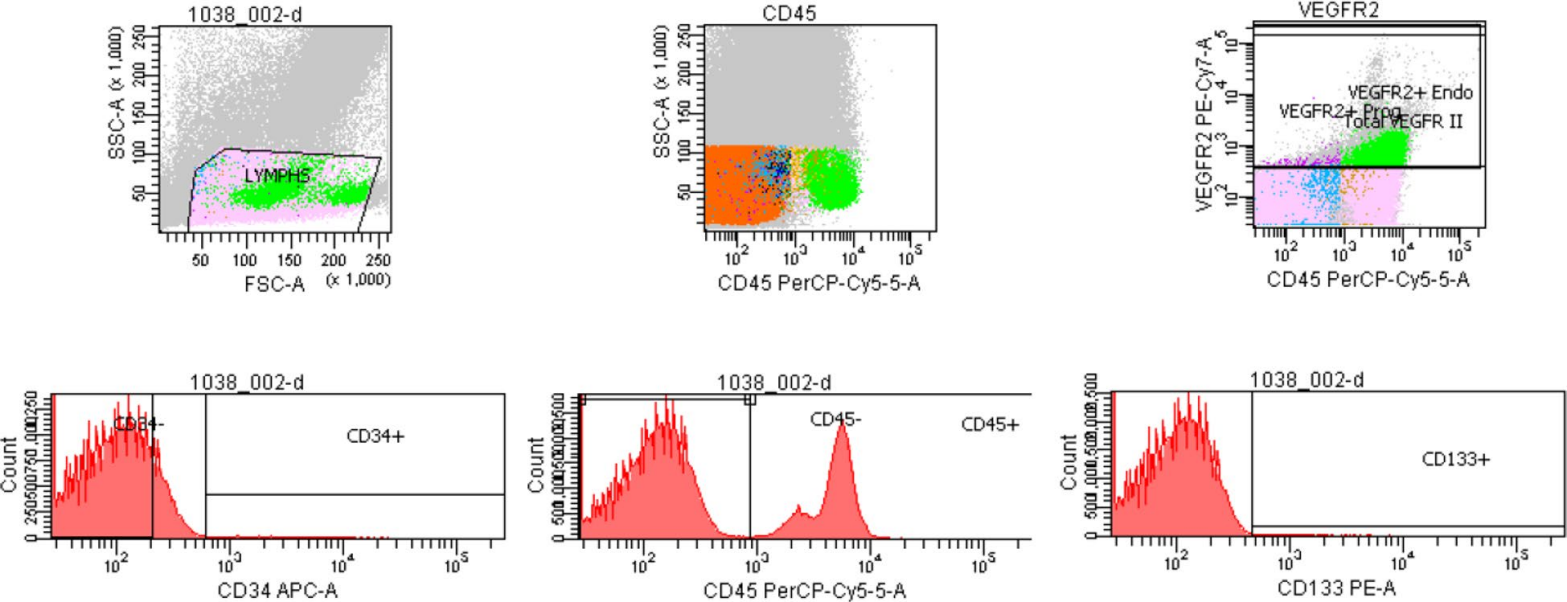
Representative dot plot FACS analysis for endothelial progenitor cells determination. Boolean analysis was performed. In all samples, the CD34 expression was dim

For evaluation of EPCs, blood samples were drawn from an antecubital or existing central vein; the first 3 ml were discarded. Venous blood was collected in acid citrate dextrose tubes of 5 ml capacity 5 min before and at the end of the NMES sessions and was processed within 3 h of collection. Blood samples were kept at 4 °C throughout the procedure. Whole peripheral blood samples were analyzed by flow cytometry [17]. During the procedure, fresh samples were centrifuged at 700 g for 20 min with no brake. The upper phase (plasma) was gently removed into a separate tube and stored in 0.25 ml aliquots. The lower phase containing the blood cells was resuspended using 10 ml of cold 1 × PBS containing 0.5 % (w/v) BSA and 1.5 mM EDTA and centrifuged at 700 g for 20 min with no brake for a second time. The upper phase was removed and discarded; the cell pellet was resuspended, and 2.5 ml was transferred into a separate tube and kept on ice. Concomitantly, 500 μl of samples was also transferred into one isotype control and three sample tubes and the appropriate antibodies were added: Then, 9 ml of ACK lysing buffer was added (to lyse red blood cells), vortexed briefly and incubated at room temperature (18–25 °C) for 3 min. It was washed twice with 9 ml of cold regular 1 × PBS and centrifuged at 250 g at 4 °C with brake for 5 min. The cell pellet was resuspended in 500 μl of 1 × PBS, and the samples were filtered through 40-μm cell strainer into 5-ml BD Falcon tubes. The tubes were held at 4 °C (or on ice) in the dark before acquisition on the flow cytometer. Flow cytometry was performed in the Flow Cytometry Core Laboratory with BDFACSCantoII (Becton–Dickinson) flow cytometer. An acquisition gate was established that included mononuclear cells but excluded most granulocytes and debris; 10^6^ mononuclear events were routinely collected to determine this population. Finally, Boolean analysis using combination of specific surface markers was applied. Different EPCs subpopulations were quantified by cytometry markers CD34^+^/CD133^+^/CD45^−^, CD34^+^/CD133^+^/CD45^−^/VEGFR_2_^+^ and CD34^+^/CD45^−^/VEGFR_2_^+^ [17, 18] (Fig. 1).

### Statistical analyses

Continuous variables are reported as mean ± standard deviation (SD). Normality of distribution was checked with Kolmogorov–Smirnov test. Student’s paired *t* test or Wilcoxon signed-rank test (in case of not normal distribution) was employed for within-group differences. Differences between subgroups were assessed with factorial analysis of variance (ANOVA) 2 × 2 (time × group). Effect size was calculated as mean of the difference/SD of the difference. Statistical significance was set to *p* < 0.05. Statistical analyses were performed with IBM SPSS 20.

## Results

Thirty-two patients were included. Demographic and clinical characteristics are presented in Table 1. No difference was observed in these characteristics between subgroups (*p* > 0.05) in terms of NMES protocols, steroids administration and sepsis severity. Concerning sepsis severity, SOFA score was higher in the higher SOFA group (*p* = 0.04) (data not shown). NMES sessions took place on 7.6 ± 0.8 days from admission.

**Table 1:** Demographic and clinical characteristics of patients included in the study

Patients included, n	32
Age (years)^a^	58 ± 14
Gender (male/female)	23/9
APACHE II score on admission^a^	21 ± 8
SOFA score on admission^a^	7 ± 3
SOFA score on session day^a^	7 ± 3
*Diagnostic category at admission*	
Post-surgery [*n* (%)]	12 (38 %)
Respiratory failure [*n* (%)]	8 (25 %)
Cardiovascular failure [*n* (%)]	4 (12 %)
Trauma [*n* (%)]	3 (9 %)
Neurological [*n* (%)]	3 (9 %)
Other [*n* (%)]	2 (6 %)
*Comorbidities*	
Cardiovascular disease [*n* (%)]	19 (59 %)
Respiratory disease [*n* (%)]	5 (16 %)
Hepatic disease [*n* (%)]	5 (16 %)
Renal disease [*n* (%)]	5 (16 %)
Diabetes mellitus [*n* (%)]	12 (38 %)
Hematological/anticoagulated [*n* (%)]	6 (19 %)
Other [*n* (%)]	21 (66 %)
*Medication on session day*	
Corticosteroids [*n* (%)]	10 (31 %)
Antimicrobials [*n* (%)]	30 (94 %)
Noradrenaline [*n* (%)]	12 (38 %)
Sedative [*n* (%)]	15 (47 %)

*APACHE* acute physiology and chronic health evaluation, *SOFA* sequential organ failure assessment

^a^Values are expressed as mean ± SD

**Fig. 2:**
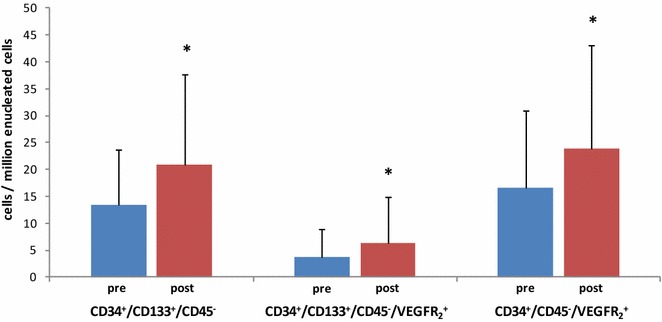
Change of the three EPCs subpopulations number (mean + SD) before and after the NMES sessions. *EPCs* endothelial progenitor cells; *asterisk* significant difference compared to pre-NMES value (*p* < 0.05)

**Fig. 3:**
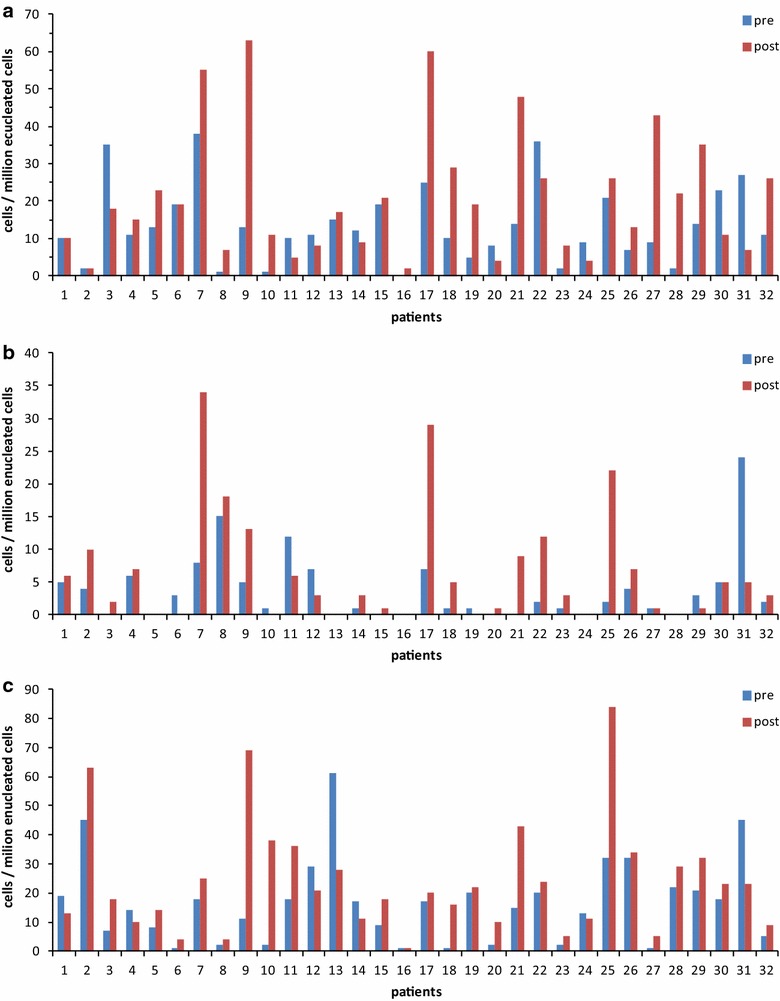
Change of EPCs number before and after the NMES sessions for each of the 32 patients included. **a** CD34^+^/CD133^+^/CD45^−^ EPCs, **b** CD34^+^/CD133^+^/CD45^−^/VEGFR_2_^+^ EPCs, **c** CD34^+^/CD45^−^/VEGFR_2_^+^ EPCs. *EPCs* endothelial progenitor cells

In the whole group, NMES increased CD34^+^/CD133^+^/CD45^−^ EPCs from 13.5 ± 10.2 to 20.8 ± 16.9 cells/10^6^ enucleated cells (+54 %, corresponding to effect size: 0.46, *p* = 0.02) (Figs. 2, 3). In regard to subgroup comparisons (Table 2), this EPCs population increased in MF (*p* = 0.04), but not in HF (*p* = 0.18). No between-protocol differences were observed (*p* = 0.60). Baseline number was lower in MF than HF (*p* = 0.03). CD34^+^/CD133^+^/CD45^−^ EPCs count also increased in the nonsteroids (*p* < 0.01), but not in the steroids group (*p* = 0.46). A significant between-group difference was observed (*p* = 0.02), while no difference was found in baseline number (*p* = 0.21). Finally, lower SOFA score (*p* = 0.04) and higher SOFA score patients (*p* = 0.07) increased CD34^+^/CD133^+^/CD45^−^ EPCs number, with no between-group difference either in total (*p* = 0.88) or at baseline (*p* = 0.95).

**Table 2:** EPCs numbers (cells/million enucleated cells, mean ± SD) before and after NMES sessions for the subgroups in terms of NMES protocol, steroids administration and sepsis severity

Subgroups	CD34^+^/CD133^+^/CD45^−^ EPCs				CD34^+^/CD133^+^/CD45^−^/VEGFR_2_^+^ EPCs				CD34^+^/CD45^−^/VEGFR_2_^+^ EPCs			
	Pre-	Post-	p*	*p* ^#^	Pre-	Post-	p*	*p* ^#^	Pre-	Post-	p*	*p* ^#^
*By NMES protocols*												
HF	18.0 ± 11.6	23.6 ± 18.1	0.18	0.60	4.8 ± 4.6	7.3 ± 9.4	0.31	0.91	15.9 ± 15.6	18.6 ± 11.6	0.13	0.20
MF	10.1 ± 7.6	18.7 ± 16.1	0.04		2.9 ± 5.6	5.8 ± 8.0	0.06		17.0 ± 14.1	28.0 ± 23.0	0.03	
*By steroids administration*												
Steroids	16.7 ± 8.8	14.2 ± 8.6	0.46	0.02	5.5 ± 7.5	2.6 ± 2.3	0.13	<0.01	21.0 ± 19.3	22.5 ± 22.5	0.84	0.21
Nonsteroids	12.1 ± 10.6	23.8 ± 19.0	<0.01		3.0 ± 3.7	8.2 ± 9.8	<0.01		14.5 ± 11.8	24.5 ± 18.1	<0.01	
*By sepsis severity*												
Lower SOFA score patients	13.4 ± 10.1	22.5 ± 20.9	0.04	0.88	3.3 ± 3.7	4.9 ± 7.7	0.50	0.43	14.9 ± 15.6	21.4 ± 17.8	0.24	0.82
Higher SOFA score patients	13.2 ± 9.6	21.4 ± 13.8	0.07		4.6 ± 6.4	8.5 ± 9.4	0.04		16.6 ± 13.1	24.7 ± 19.9	0.01	

*EPCs* endothelial progenitor cells, *HF* high-frequency NMES protocol, *MF* medium-frequency NMES protocol

* *p* value for within-group differences; ^#^ *p* value for between-group differences

Similar results were observed for CD34^+^/CD133^+^/CD45^−^/VEGFR_2_^+^ EPCs, which were increased in the whole cohort from 3.8 ± 5.2 to 6.4 ± 8.5 cells/10^6^ enucleated cells (+68 %, corresponding to effect size: 0.33, *p* = 0.04) (Figs. 2, 3). In relation to subgroup comparisons (Table 2), these were tended to increase in MF (*p* = 0.06), but not in HF (*p* = 0.31), while no between-protocol difference was found (*p* = 0.91). CD34^+^/CD133^+^/CD45^−^/VEGFR_2_^+^ EPCs count increased in the nonsteroids (*p* < 0.01), but not in the steroids group (*p* = 0.17); a significant between-group difference was observed (*p* < 0.01). Finally, higher SOFA score (*p* = 0.04) but not lower SOFA score patients (*p* = 0.50) increased CD34^+^/CD133^+^/CD45^−^/VEGFR_2_^+^ EPCs number, with no between-group difference (*p* = 0.43). In addition, no differences between subgroups at baseline were found for all comparisons employed (*p* > 0.05).

In relation to CD34^+^/CD45^−^/VEGFR_2_^+^ EPCs, the entire group increased number from 16.5 ± 14.5 to 23.8 ± 19.2 cells/10^6^ enucleated cells (+44 %, corresponding to effect size: 0.42, *p* < 0.01) (Figs. 2, 3). Its count did not change in HF (*p* = 0.13), while it increased in MF (*p* = 0.03). It was also increased in the nonsteroids (*p* < 0.01), but not in the steroids group (*p* = 0.84). Finally, higher SOFA score group (*p* = 0.01), but not lower SOFA patients (*p* = 0.24), increased CD34^+^/CD45^−^/VEGFR_2_^+^ EPCs number. No significant between-group difference was found in terms of NMES protocol (*p* = 0.20), steroid administration (*p* = 0.21) or sepsis severity (*p* = 0.82) (Table 2).

In the whole group during the NMES sessions, there were increases in heart rate (from 89 ± 20 to 92 ± 19 beats/min, *p* = 0.04) and respiratory rate (from 21 ± 6 to 22 ± 6 breaths/min, *p* = 0.03), while mean arterial pressure did not change (from 89 ± 11 to 92 ± 17 mmHg, *p* = 0.19). Increases were also observed in creatine phosphokinase (from 498 ± 961 to 526 ± 1011 IU/L, p < 0.01) and lactate (from 1.1 ± 0.4 to 1.4 ± 0.6 mmol/L, *p* < 0.01). No ECG and hemodynamic derangements occurred during the sessions.

## Discussion

This study showed that a single NMES session increased counts of endothelial progenitor cells in critically ill patients with sepsis. To the best of our knowledge, this is the first study to demonstrate NMES efficacy on mobilizing progenitor cells, an index of the endothelium restoration potential, in ICU patients.

Previous pilot studies, exploring the effects of short-term protocols employing NMES in combination with intermittent hypobaric hypoxia or exercise, have shown increased number of progenitor cells in healthy subjects and patients with traumatic brain injury [19, 20]; obviously, these studies are not feasible to compare with our study.

In ICU patients, NMES seems to prevent muscle weakness and improve muscle strength [8, 9] while it has also the potential to prevent muscle atrophy [7, 10, 21]. Acutely applied NMES can induce systemic beneficial effects on microcirculation, which is related to mechanisms of ICU-acquired weakness, including disturbances in macro- and microvascular function [22, 23]. Further evidence suggests that NMES can reduce ICU-acquired weakness incidence and duration of weaning, although this needs to be verified by large RCTs [9, 24]. From these points of view, NMES has been suggested as a form of exercise to mobilize and induce rehabilitative interventions to ICU patients [25]. There is also increasing interest in the NMES role concerning therapy of pressure ulcers [26] and prevention of deep vein thrombosis [22, 23].

Previous studies have also demonstrated increased levels of EPCs subpopulations or their migratory capacity in response to acute bouts of aerobic exercise in healthy populations and other critically ill patients, such as patients with CHF [5, 14]. These effects were attenuated in CHF [27]. Interestingly, recent data also suggested a beneficial effect of acute resistance training on improving EPCs counts and angiogenic factors in healthy subjects [13]. NMES, as applied in this study, may have more similarities to resistance exercise in that it stimulated isolated muscle groups.

Shear stress may be suggested as a triggering factor for EPCs release after a single NMES session. Shear stress-induced up-regulation and increased activity of endothelial nitric oxide synthase are related to increased nitric oxide production by endothelial cells [28], contributing to amplified number and activity of circulating EPCs [3]. NMES application on the calf muscles of healthy subjects and patients after total hip replacement surgery acutely increased popliteal blood flow and volume flow, reflecting increases in shear stress-induced vasodilatation [22, 23]. The importance of ischemic/hypoxic stimulus to increase EPCs count has been also implied in short-term studies enrolling patients with cardiovascular disease [29]. In a recent animal study, short-term NMES-induced ischemic training moderately increased EPCs [30]. EPCs have been also increased in response to acute exercise- and non-exercise-induced ischemic stimulus in patients with peripheral arterial occlusive disease and healthy participants, respectively [31]. NMES, mainly resulting in isometric contractions, has the potential to induce hypoxic stimuli, as suggested by alterations in microcirculation indices during NMES sessions in healthy populations [32] and ICU patients [16]. These mechanisms may relate to up-regulation of transcriptional factors, such as matrix metalloproteinases, stromal cell-derived factor 1 and vascular endothelial growth factor, which mediate processes to promote proliferative and migratory capacities of circulating EPCs [5, 13, 31]. The proposed mechanisms and mediating factors in relation to the different subpopulations of progenitor cells, as well as the time course of the changes, should be addressed in future studies exploring the effects of NMES in ICU patients.

An interesting finding was that patients on corticosteroids did not increase progenitor cells in contrast to patients not on steroids. Corticosteroids may affect EPCs mobilization and activity. Previous studies on EPCs mobilization and function employing patients on corticosteroids medication have provided similar [33, 34], neutral [35] or contrasting results [36, 37]. In the latter studies, this finding could be related to the fact that steroids administration had a stronger effect on interleukin 1 reduction and rheumatic disease remission—the main cause of progenitor cells suppression—than EPCs number per se [38]. In this study, no difference was detected between the two corticosteroids subgroups at baseline EPCs counts and that was also the case for SOFA and APACHE scores. Several confounding factors such as small sample size, severity of disease (not able to be reflected on conventional scales, such as SOFA score) and presence of inflammation may have interfered with these observations. Further research is necessary to explore the role of corticosteroids on mobilizing EPCs in response to NMES before reaching definite conclusions.

Both medium- and high-frequency protocols were found to similarly increase EPCs. Although MF, but not HF, was found to amplify EPCs count, this may be related to the sample size, since no interaction effect was observed. Another confounding factor might also be the distribution of sedated and awake patients in the NMES protocol subgroups, resulting in differences related to current tolerance and evoked muscle contraction. In a previous study of our group employing similar NMES protocols, both currents were equally effective in improving thenar oxygen consumption rate, endothelial reactivity and vascular reserve as well as oxygen consumption, demand and supply at the stimulated muscle, measures of systemic and local microcirculation, respectively. These improvements were well correlated to the strength of contraction [16]. Interestingly in another study, oxygen consumption in the biceps brachii during isometric contraction—which could be considered to reflect the magnitude of hypoxic stimuli of the working muscle—increased in proportion to the force developed [39]. In this study, although muscle strength contraction was not systematically recorded, some patients were hardly responded to NMES (no contraction at all or just palpable contraction), as previously observed [40]; however, all patients, ‘responders’ and ‘non-responders,’ were included in the analysis, which, in turn, might be related to the marked variability observed among patients. The optimal NMES characteristics to apply in ICU patients are still under investigation [8]. Future studies should further explore the role of NMES to mobilize progenitor cells in relation to strength contraction of the stimulated muscle.

Although ICU patients most to benefit from NMES application have not yet clearly determined, septic patients are definitely a target group of interest. Sepsis induces endothelial and microcirculation dysfunction and is a risk factor for multi-organ failure and ICU-acquired weakness [1, 41]. Endothelial restoration capacity is impaired in sepsis, and circulating EPCs have been suggested to inversely associate with SOFA score and organ dysfunction [42]. Recently, exogenously administered EPCs in septic mice increased anti-inflammatory mediators, attenuated liver and kidney injury, augmented markers of endothelial cell function/inflammation and EPCs recruitment, and improved survival [43]. In this study, both SOFA-based severity subgroups increased EPCs, likely implying that NMES has the potential to mobilize progenitor cells in septic patients irrespective of severity status; however, the clinical implications of this finding need to be explored.

In this study, small—although significant—increases in heart rate, respiratory rate, creatine phosphokinase and lactate were also observed due to NMES application. Interestingly, progenitor cells mobilization after acute exercise was recently related to creatine phosphokinase as a marker of ‘muscle damage’; whether the increase seen in this study might have a role, remains to be elucidated [12]. Overall, these increases, seemingly having not any clinical significance for ICU patients, are rather reflective of a systemic effect of NMES, in line with previous findings from our group [11, 16]. The absence of any clinically significant side effects in terms of cardiac/hemodynamic derangements and ‘muscle damage’ further emphasizes on the NMES role as a safe and well-tolerated intervention in critically ill patients.

On the whole, our findings provide evidence that NMES mobilizes EPCs in ICU patients, measures of the endothelial restoration potential. Increased mobilization of EPCs was approached in terms of counts elevation rather than functional improvement; since increased EPCs counts do not necessarily reflect elevated function, future studies should also address NMES efficacy to affect functional aspects of these cells categories. Accumulating evidence also supports the significance of NMES concerning prevention of ICU-acquired weakness, as well as its potential role in the prevention of thromboembolism and pressure ulcers, comorbidities of critical illness related to disturbed endothelial function. The relation of NMES-induced EPCs mobilization to prevention of these comorbidities warrants further investigation.

A limitation of the study was the small sample size of the patients included; comparisons between or within subgroups were, in some instances, underpowered to demonstrate differences in progenitor cells alterations. In addition, strength contraction and current intensities were not systematically recorded, which could possibly allow for further insight into the potential mechanisms of EPCs mobilization; however, by design, current intensities applied—optimally aiming at full muscle contraction—were the maximal tolerated by non-sedated and sedated patients, implying maximum applicable contraction in each case.

## Conclusion

NMES acutely mobilized EPCs in severe ICU patients. These effects were not dependent on NMES protocol or sepsis severity status, while they were observed to relate to corticosteroids administration. These later findings the NMES-induced mechanisms of improved EPCs mobilization and their clinical consequences warrant further investigation.

## Abbreviations

EPCs: endothelial progenitor cells
CHF: chronic heart failure
NMES: neuromuscular electrical stimulation
HF: high-frequency protocol
MF: medium-frequency protocol
SD: standard deviation
APACHE: acute physiology and chronic health evaluation
SOFA: sequential organ failure assessment
